# Preserving replication fork integrity and competence via the homologous recombination pathway

**DOI:** 10.1016/j.dnarep.2018.08.017

**Published:** 2018-11

**Authors:** Anissia Ait Saada, Sarah A.E. Lambert, Antony M. Carr

**Affiliations:** a Institut Curie, PSL Research University, CNRS, UMR3348, F-91405, Orsay, France; b University Paris Sud, Paris-Saclay University, CNRS, UMR3348, F-91405, Orsay, France; c Genome Damage and Stability Centre, School of Life Sciences, University of Sussex, Falmer, Sussex, BN1 9RQ, UK

**Keywords:** Replication stress, Recombination, Genome instability, Fork integrity, Fork restart

## Abstract

Flaws in the DNA replication process have emerged as a leading driver of genome instability in human diseases. Alteration to replication fork progression is a defining feature of replication stress and the consequent failure to maintain fork integrity and complete genome duplication within a single round of S-phase compromises genetic integrity. This includes increased mutation rates, small and large scale genomic rearrangement and deleterious consequences for the subsequent mitosis that result in the transmission of additional DNA damage to the daughter cells. Therefore, preserving fork integrity and replication competence is an important aspect of how cells respond to replication stress and avoid genetic change. Homologous recombination is a pivotal pathway in the maintenance of genome integrity in the face of replication stress. Here we review our recent understanding of the mechanisms by which homologous recombination acts to protect, restart and repair replication forks. We discuss the dynamics of these genetically distinct functions and their contribution to faithful mitoticsegregation.

## 1 Introduction

Genome duplication is a highly controlled process organized in three main steps: the firing of replication origins to initiate DNA synthesis; elongation by replication forks that progress bi-directionally from origins to copy the DNA and termination, when converging forks meet and fuse (reviewed in [1]). Eukaryotic genomes contain multiple replication origins, the firing of which is spatially and temporally regulated (reviewed in [2]). During an individual S phase, only a subset of the potential origins are instrumental in genome duplication. The remaining dormant origins are passively replicated by forks emerging from neighboring active origins. The resources necessary for DNA synthesis, such the availability of dNTPs and core replication factors, require a careful balance between initiation and elongation [3,4]. Excessive origin firing is detrimental to genome maintenance because it results in the exhaustion of replication factors. Conversely, the slow progression of replication forks provides a window of time for dormant origins to fire, promoting the completion of DNA replication before cells enter mitosis in order to avoid mitotic abnormalities and the transmission of damage to the next generation.

**Fig. 1:**
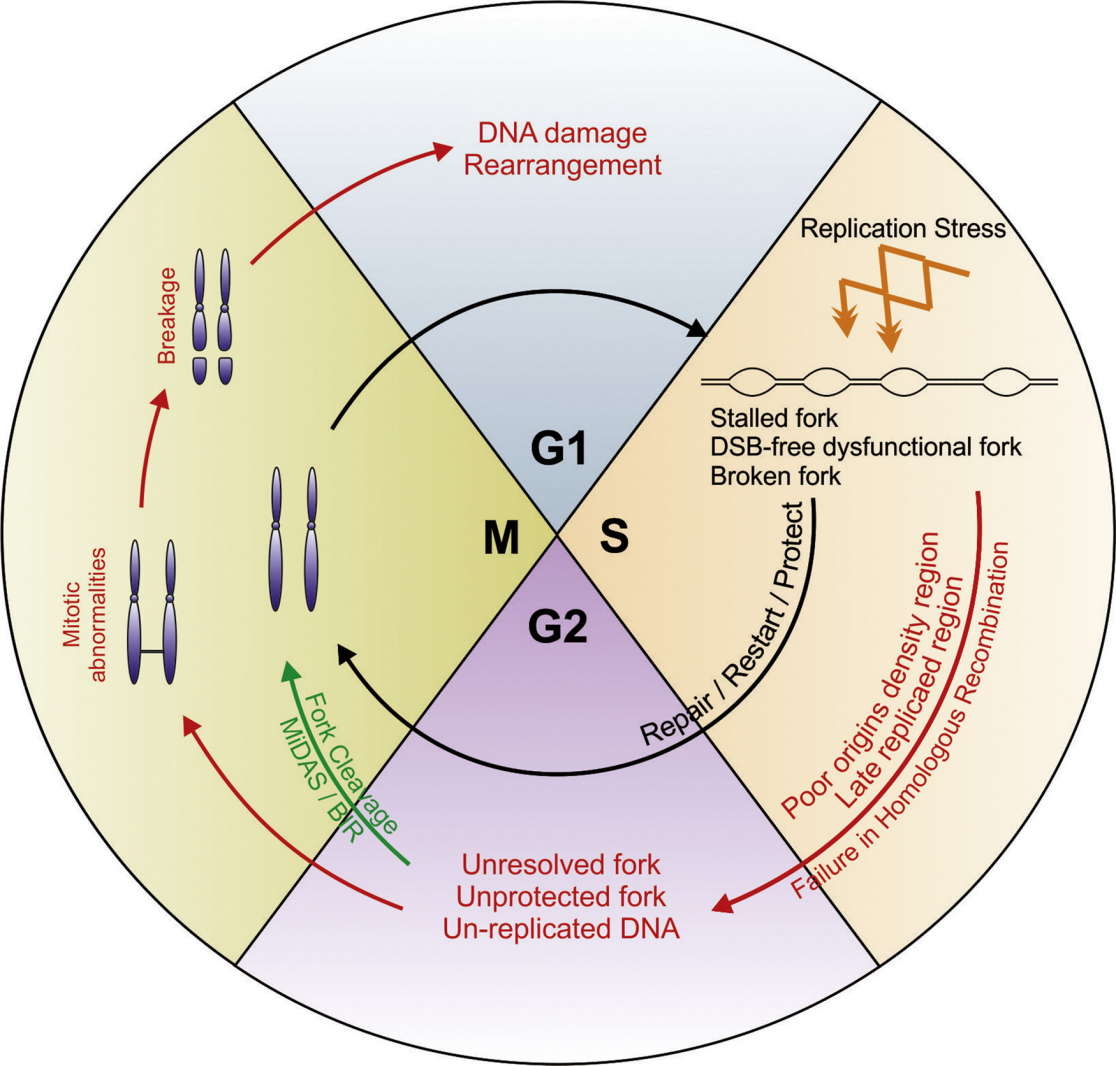
**Pathways preventing transmission of DNA damage upon replication stress**. Replication stress results in various types of corrupted replication forks. These include stalled forks that retain their replication competence and dysfunctional fork that have lost their replication competence. This later class can either be associated with a double strand break (broken fork) or be DSB-free. Sub-pathways of homologous recombination (HR) act to protect stalled forks from becoming dysfunctional, or restart and repair dysfunctional forks. In this way recombination factors ensure either successful merger with a converging fork (fork protection), or promote recombination-dependent replication (RDR) to allow replication to be completed when a converging fork is not available. HR therefore promotes the completion of DNA replication in a timely manner, avoiding mitotic catastrophe. When HR is genetically impaired (*i.e.* by mutations in HR genes) late replicated regions and/or regions with low origins densities can accumulate unprotected forks, unresolved replication intermediates and un-replicated DNA. These may persist through late G2 and into mitosis. Fork cleavage by structure-specific endonucleases (*i.e.* Mus81) offers the opportunity to resolve replication problems via break-induced replication (BIR) that results in mitotic DNA synthesis (MiDAS). The persistence of abnormal replication intermediates in mitosis jeopardizes faithful chromosome segregation, resulting in various types of mitotic abnormalities (*i.e.* chromatin bridges, ultra-fine bridges, lagging chromosomes and micronuclei). Mitotic abnormalities can trigger chromosomal breakage and rearrangement which are transmitted to the next generation.

Alterations to the dynamics of genome duplication can lead to replication errors that cause genome instability, from the nucleotide level up to large-scale chromosomal rearrangements (reviewed in [5]). Such changes to DNA replication are collectively known as replication stress (Fig. 1), a term that covers a wide variety of situations, including intrinsic fork obstacles (i.e. DNA bound proteins and DNA-RNA hybrids), DNA damage, secondary DNA structures, deficiency or excess of origin firing and replication occurring in inappropriate metabolic conditions (for example, without fully coordinated regulation of the cell cycle - often referred to as unbalanced DNA replication). The majority of these events result in alterations to replication fork progression and challenge the faithful duplication of the genome (reviewed in [6,7]).

Slow or arrested replication forks stimulate the use of potentially mutagenic DNA repair pathways that can lead to the accumulation of mutations and chromosomal rearrangements (reviewed in [8,9]). In addition, if the slow progression of replication cannot be compensated by the firing of dormant origins, there is the risk that cells will enter mitosis with unresolved replication intermediates (Fig. 1). This is exemplified at common fragile sites (CFS), which are defined as being prone to DNA breakage in mitosis (reviewed in [2]). CFS are often replicated late in S phase and/or associated with origin-poor genomic regions. When un-replicated loci segregate, the intertwined DNA strands in the un-replicated region form an ultra-fine bridge (UFB) because each strand of the duplex belongs to a separate sister chromatid [[10], [11], [12], [13]]. Thus, the DNA is stretched during mitosis. CFS-associated UFBs are refractory to conventional staining with DNA intercalating agents and are rapidly coated by proteins, including PICH (Plk1-intercation checkpoint helicase) and the single stranded DNA binding protein RPA. UFBs resulting from the segregation of un-replicated chromosome regions can be distinguished from other UFBs (for example those associated with topological entanglement at centromeres) because they are flanked by two sister FANCD2 (Fanconi anemia group D2) foci (reviewed in [14]). Thus, replication stress favors the formation of a subclass of UFBs that are likely to break during mitosis, generating DNA lesions that are transmitted to the two daughter cells [[15], [16], [17], [18]].

The current interest in understanding the cause, and biological consequences of, replication stress derives from observations that replication stress frequently accompanies pathological and physiological processes. For example, endogenous replication stress has been demonstrated to be a common source of genome instability in pre-neoplastic lesions, contributing significantly to the acquisition of the multiple genetic modifications that define carcinogenesis [19,20], reviewed in [21]. Known as "oncogene-induced replication stress" (OIS), this is thought to arise because the initial activation of an oncogene interferes with the DNA replication program, forcing cells to initiate DNA synthesis in inappropriate metabolic conditions. These may include insufficient dNTP pool, insufficient or excessive licensed origins, conflicts with the transcription program and replication in the presence of inappropriately active nucleases due to cell cycle dysregulation [[22], [23], [24], [25], [26]]. Unbalanced DNA replication due to OIS causes cells to accumulate mutations in S phase and to enter mitosis with persistent and/or unusual replication intermediates (Fig. 1). The processing of such intermediates can also contribute to oncogene-induced genotoxicity [27]. At later stages of carcinogenesis, replication stress has also been linked to structural and numerical chromosomal instability (CIN) that fuels the process of metastasis [28,29]. In addition to contributing to carcinogenesis through inducing mutations and chromosome instability, replication stress has also been proposed to represent an Achilles heel of cancer cells that can be chemically targeted for anti-cancer therapy [30].

Replication stress has also been linked to normal physiology: embryonic stem cells (ESCs) and pluripotent stem cells (PSCs) are characterized by rapid proliferation and constitutively express hallmarks of replication stress. These include the presence of DNA repair foci in unperturbed cells, reduced DNA replication fork speed and the excess accumulation of single stranded DNA at replication forks [31]. Cellular differentiation alleviates the expression of these replication stress markers. Since the endogenous replication stress observed in old hematopoietic stem cells impacts on their self-renewal capacity [32], replication stress is proposed to underlie the functional decline of PSCs. Induced PSCs (iPSCs), derived by reprogramming adult somatic cells, also exhibit replication stress and associated genome instability, compromising their use in regenerative medicine [33]. Lowering replication stress using chemical tools has therefore been proposed as one strategy to optimize the use of PSCs and iPSCs in regenerative medicine.

Because the progression of replication forks is an intrinsically precarious process, replication forks need to be well-escorted in order that they can appropriately react to obstacles. When a replication fork transiently stalls, is subjected to prolonged arrest or collapses to become dysfunctional, it is likely to be rescued by a converging fork, the arrival of which is favored by the activation of a nearby dormant origin. When a converging fork is not available to resolve the problem caused by a stalled dysfunctional fork (for example when two converging forks collapse in a region with no intervening replication origin), the dysfunctional fork must be restarted if the cell is to avoid trying to separate un-replicated chromosome regions. Importantly, these two fork rescue pathways, fork-restart and rescue by a converging fork, are in dynamic competition. The restart of a replication fork can be initiated and then aborted because of the arrival of an opposite fork. Recently, it has been revealed that these two fork rescue pathways require distinct functions of the homologous recombination pathway, which are discussed below [34].

In many experiments where cells are treated with agents that cause genome-wide replication stress, it is difficult to know if the individual affected forks are functional or dysfunctional. For example, the initial cellular response to inhibition of DNA polymerases (for example using hydroxyurea to deplete dNTP pools or aphidicolin to directly inhibit polymerization) is activation of the intra-S phase checkpoint. This pathway attempts to maintain the stalled or slow fork in a replication competent state and, depending on the origin of the fork obstacle, collaborate with a variety of accessory DNA helicases and DNA repair factors to aid the timely resumption of fork elongation. If successful, such replication competent forks simply resume canonical DNA replication. For those forks that do become dysfunctional (and the proportion of these is expected to increase over time), it is unclear what happens both at the level of fork DNA structure and the association of replication proteins. When forks become dysfunctional, helicases and repair factors preserve the integrity of the fork to facilitate either its fusion with a converging fork or, less frequently, to restart replication in order to complete S phase. These processes utilize the homologous recombination (HR) pathway and are discussed in more detail below.

## 2 The HR pathway and replication stress tolerance

HR is an evolutionarily conserved DNA repair pathway whose pivotal player is the recombinase RecA in prokaryotes and Rad51 in eukaryotes (reviewed in [35]). HR is involved in the repair of several types of DNA lesions, including Double Strand Breaks (DSBs) and single stranded DNA gaps (ssDNA gaps). Research in prokaryotes first established that HR is intimately coupled to the process of DNA replication, escorting the progressing replication forks to protect and, if necessary, repair and restart them. Subsequently, HR has also been shown to be a key pathway that allows eukaryotic cells to tolerate replication stress [[36], [37], [38], [39]]. Thus, HR is believed to have therapeutic relevance to anti-cancer therapy.

RecA and Rad51 exhibit many biochemical properties in vitro, the regulation and biological functions of which are not fully elucidated in vivo (reviewed in [40]). Both proteins bind ssDNA and, to a lesser extent, dsDNA. The binding to ssDNA requires RecA or Rad51 to bind ATP and is cooperative, allowing a nucleoprotein filament to form around ssDNA. Eukaryotic Rad51 has poor ATPase activity but, upon ATP hydrolysis, Rad51 loses affinity for ssDNA. Following ATP binding and filament formation, Rad51 promotes pairing between homologous DNA molecules, strand invasion and strand exchange. In vivo, the Rad51-filament is thought to be the active recombination intermediate driving the homology search, the subsequent pairing of homologous sequences and strand invasion step of recombination.

In vivo, the activity of eukaryotic HR has been most extensively studied in the context of DSB repair (reviewed in [41]). Recombinational repair of DSBs is initiated by the 5′-3′ resection of DSB ends to expose a ssDNA with 3′ ends that are coated with RPA. The subsequent formation of stable Rad51 filaments on these substrates requires numerous HR mediators. The main mediator in yeast models (*S. cerevisiae* and *S. pombe*) is Rad52, which is required to displace RPA and facilitates loading of Rad51. Yeast Rad52 also possesses a separate single strand annealing activity that can mediate Rad51-independent recombination events: Rad52 promotes the pairing of two complementary ssDNA that are coated with RPA. In metazoans, the key Rad51 loader is BRCA2 rather than Rad52. Heterozygous mutation of BRCA2 predisposes patients to ovarian and breast cancers. BRCA2 is not structurally related to Rad52, has no single strand annealing activity and is not able to displace RPA from ssDNA. This last function is achieved by DSS1, a BRCA2 interacting factor, which mimics DNA to facilitate RPA displacement [42]. Metazoans do contain a structural homolog of Rad52 that plays a role in tolerating replication stress, but this function appears to utilize the single stand annealing activity of Rad52 and is reported to be independent of Rad51 [43,44].

**Fig. 2:**
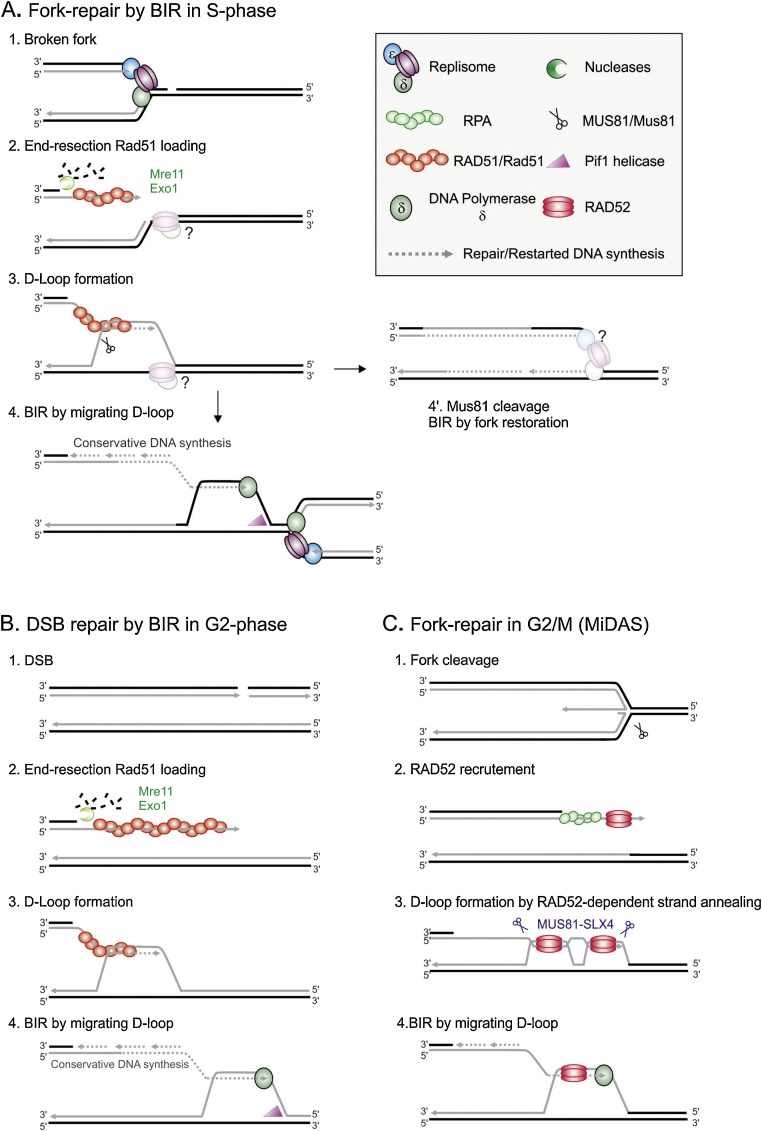
**Models of DSB-initiated recombination-dependent replication**. A. Replication forks encountering a DNA nick are converted into broken forks, which may be accompanied by the loss of replisome components (1). The DNA end-resection machinery (*i.e.* Mre11 and Exo1) generates a single-stranded 3′overhang that is coated by the RAD51 recombinase (2) which promotes strand invasion into the sister chromatid to form a D-loop structure from which DNA synthesis can be primed (3). In budding yeast, break-induced replication (BIR) proceeds by conservative DNA synthesis using a migrating D-loop that is mediated by the Pif1 helicase. The non-essential Pol32 sub-unit of the DNA polymerase delta is required for BIR, which is highly error-prone and limited by an incoming converging fork (4). Alternatively, Mus81 endonuclease can cleave the D-loop structure allowing the restoration of semi-conservative DNA synthesis (4′). It is not known if the replisome associated with such a re-set fork is canonical or not. B. BIR can be initiated by the breakage of a single chromatid in G2 (1,2). The migrating D-loop and its associated conservative DNA synthesis can proceed until the end of the chromosome (3). In the example shown, the sister chromatid provides the donor template, but BIR can employ ectopic homologous sequence during repair of a DSB. BIR in G2 generates long stretches of ssDNA (4) which is highly sensitive to mutations and formation of secondary recombination intermediates. C. Unresolved replication forks in mammalian cells are cleaved by MUS81 in late G2 and mitosis (1). The strand annealing activity of RAD52 (2) promotes the formation of joint-molecules (3) the nature of which remains elusive. Mitotic DNA synthesis (MiDAS) requires POLD3, a component of the DNA polymerase delta homologous to yeast Pol32. Here, the sister chromatid is shown as the donor template, but MiDAS can also result in ectopic micro-homology mediated BIR (MMBIR).

In mammalian cells the Rad51 pathway of HR is not active in G1 [45,46]. In S phase and G2, once the Rad51 nucleoprotein filament is formed it promotes a homology search and invades a homologous duplex (usually this target sequence is the sister chromatid) to form a three-stranded paranemic intermediate. The invading strand then displaces one of the strands of the recipient duplex and anneals with the complementary strand. The non-complementary strand is displaced as a ssDNA coated with RPA. The resulting structure is called a displacement loop (D-loop). The 3′ end of the invading strand is capable of priming DNA synthesis, thus promoting repair of the DSB. At a dysfunctional replication fork, the formation of a D-loop would provide an appropriate opportunity to re-prime DNA synthesis (Fig. 2A). While the D-loop is formed during DSB repair from a processed DNA end, there is accumulating evidence that the biological functions of HR at replication forks can occur independently of DSBs formation by fork cleavage. Indeed, at a replication fork, the two sister chromatids are physically associated, promoting any HR events to the appropriate template. Cleaving such a structure would likely increase the potential for ectopic recombination, an important consideration for the highly repetitive genomes of metazoan organisms

## 3 Repairing replication forks by break-induced replication

In prokaryotes replication generally initiates at a single origin and two forks replicate an entire circular chromosome (reviewed in [47]). Thus, replication is largely unidirectional within each half of the chromosome. Because of this, a dysfunctional fork is not usually rescued by a converging fork. Replication is therefore restarted at dysfunctional forks by recombination-dependent replication (RDR). To effect RDR, a dysfunctional fork is, if not already broken, first cleaved to form a single-ended DSB that is rapidly processed to ssDNA and coated with a RecA a filament that subsequently invades the template duplex to establish a D-loop. The resulting DNA structure is rapidly processed by nucleases and the resulting RDR fork appears to be canonical, with the replicative helicase being reloaded by a specialized machinery.

In eukaryotic organisms, a single-ended DSB can also initiate replication, but the mechanism is somewhat different to that in prokaryotes, most likely because the necessary loading of the Cdc45-MCM-GINS (CMG) replicative helicase outside of G1-S phase would conflict with the mechanism required to ensure a single round of replication per cell cycle [48]. In addition, the requirement for RDR in eukaryotes is much reduced since the majority of dysfunctional forks are rescued by a converging fork derived from a nearby origin of replication. Budding yeast has been extensively used to characterize replication events induced by a DSB end (known as BIR; break-induced replication, reviewed in [49]). The experimental system used involves generating a single site-specific enzymatic break in G2 cells (note: both sister chromatids are cleaved and thus the sister chromatid cannot act as a template for strand invasion) where only one end of the break has homology to a separate template chromosome. Thus BIR, as generally characterized, is focused on RDR initiated in G2, where the template is on a separate chromosome.

The initiation of BIR in budding yeast can follow at least two pathways: Rad51 (the eukaryotic orthologue of RecA) is required for the most common pathway (Fig. 2B). This requires a region of homology in excess of 70 bp [[50], [51], [52]]. In G2 cells there is a delay of several hours between strand invasion and the initiation of DNA synthesis within the D-loop [53], most likely caused by activation of a checkpoint that monitors second end capture (an ill defined pathway that ensures both ends of a DSB engage in DSB repair [54]). In the absence of Rad51, a second pathway that uses the strand annealing activity of Rad52 can initiate replication [55]. This Rad51-independend BIR pathway, which is generally suppressed in the presence of Rad51 [50], can be initiated at shorter regions of homology (microhomology and regions of homeology such as those present in Ty elements of budding yeast) and is thus more prone to generate gross chromosomal rearrangements [56]. Rad51-independet BIR is initiated very late, likely after the cell has attempted mitosis one or more times and secondary damage is caused by aberrant chromosome fusions and segregation.

Once established, BIR forks are not canonical and replication proceeds via a migrating D-loop [[57], [58], [59]] that can travel for over 100 kb, a form of replication that results in conservative (as opposed to canonical semi-conservative) DNA replication (Fig. 2B). While the precise architecture of the machinery driving BIR is not known, it requires various helicases such as Pif1 and Sgs1, leading and lagging strand synthesis are not coupled [58], the inessential polymerase delta accessory factor Pol32 is required [51] and the majority of the DNA is synthesized by polymerase delta. Although there are conflicting reports as to the requirement for the CMG replicative helicase [59,60], intuitively it is hard to envisage how this specialized machine would be required for D-loop migration and it is also unlikely that G2 cells would attempt to load this key marker of replication competence because this would counteract the complex processes that ensure a single round of replication in each cell cycle.

An important feature of BIR is the high level of mutations produced during replication. These arise through various processes including replication fork slippage, template exchange events and erroneous base substitutions that are inefficiently corrected by mismatch repair [58,61,62]. In addition, the newly synthesized strand is present in the cell for an extended period as single stranded DNA because leading and lagging strand synthesis are not coupled. This can result in two additional issues; firstly, newly replicated bases on the single-stranded leading strand are more susceptible to modifications that cannot be repaired in the usual fashion from an opposing duplex strand [58,63]. Second, extensive ssDNA attracts Rad51 and this can result in promiscuous strand invasions that must be kept in check by anti-recombinases such as Srs2 [64]. Further genomic instability results from the potential for loss of heterozygosity associated with copying the homologous chromosome and by the resolution of the D-loop junction (most likely when BIR is paused) by structure -specific nuclease to cause "half-crossovers" [65,66].

While BIR has been extensively studied in G2/M phase in budding yeast, a recent study examined BIR in budding yeast S phase cells by using a nickase to introduce a site-specific persisting single strand nick that is processed to a DSB when encountered by the replication fork [67] (Fig. 2A). This identified some significant differences between the two cell cycle stages: while replication was restarted in S phase in a Rad51-dependent manner and was highly error prone, initiation occurred without the two hour delay associated with the second-end capture checkpoint and Mus81 activity limited the length of DNA that was replicated in a highly error prone manner. This is most likely because the D-loop was being "reset" into a more fork-like structure. It was not clear if replication continued after this Mus81 activity in a less error-prone manner or simply stopped, allowing the downstream sequences to be replicated by an incoming converging fork. As expected (and which was shown previously [68,69] using a DSB-free recombination-dependent replication system in fission yeast – see below), it was also noted that incoming canonical forks rescued the increased mutation frequency associated with BIR in S phase. These differences may reflect the physiological role of BIR in S phase: the rescue of dysfunctional broken replication forks that are not resolved by converging fork. It is possible that BIR in G2/M cells is simply a consequence of failed homologous recombination (i.e. a pathological response to failed second end capture). However, it may have a physiological function in rescuing DSBs introduced within or close to telomeres, where one chromosome fragment can be lost due to its small size.

BIR in metazoan cells has been implicated in restarting DNA replication during S phase in Xenopus in response to DSBs introduced specifically at replication forks [70] and in human cells at fragile sites or when they are mutated in some key homologous recombination proteins such as BRCA2 [44,71,72]. It has also been proposed to play a role in telomere maintenance in ALT (alternative lengthening of telomeres) cells [[73], [74], [75]]. The error prone nature of BIR has thus been proposed to underpin a wide range of genetic lesions associated with human disease and cancer [43,62,76,77]. The formal definition of BIR - replication initiated from a DNA double strand break - may not always apply to recombination dependent DNA synthesis in mammalian cells, which can also be initiated from single strand lesions associated with dysfunctional replication forks (see below). Indeed, there is circumstantial evidence that the first response to a dysfunctional mammalian replication forks is not associated with DSB formation. DSBs arise later, possibly providing an alternative opportunity to rescue the situation [78,79]. Unfortunately, the nomenclature used across the field regarding the status of a replication fork is not consistent and in many experiments it is not possible to clearly distinguish between arrested forks that remain competent to resume and dysfunctional forks that would first need to be restarted (likely by HR-based mechanisms). In addition, there is a tendency to assume that RDR in metazoans is always initiated through a DSB intermediate, so BIR is often considered synonymous with RDR in eukaryotes. These issues cause confusion, prevent a clear distinction between the metabolism of stalled (replication competent) and dysfunctional forks, and encourage the assumption that recovery of all forms of dysfunctional forks can essentially be considered as a single pathway.

However, Rad51-dependent RDR in human cells has clear links with genome rearrangements that occur between repeated sequences due to ectopic recombination and the many microhomology-mediated events observed in human genetic disorders and cancer cells [21,80,81]. Micro-homology-mediated rearrangements have been attributed to a model known as micro-homology-mediated break induced replication (MMBIR [[82], [83], [84]], which is proposed to be Rad51-independent and equivalent to the Rad51-independent BIR characterized in *S. cerevisiae*. However, it remains unclear how distinct MMBIR is from Rad51-dependent RDR, since the template switching that occurs during Rad51-dependent RDR (BIR) - also related to the Fork Stalling and Template Switching model (FoSTeS [85]) - could explain some, if not the majority, of the rearrangements attributed to MMBIR.

Recent work has identified mitosis-specific DNA synthesis (MiDAS, Fig. 2C) in human cells [72,86]. MiDAS is conservative, reportedly Rad51 independent and Rad52 dependent [43,44]. MiDAS has been shown to occur at common fragile sites and at telomeres in cancer cells that maintain telomeres by ALT. Interestingly, MiDAS acts at CFS to suppress the formation of ultrafine bridges, and may represent a final opportunity to complete replication. MiDAS is initiated by the cleavage of persistent arrested fork structures by Mus81 and Slx4 [44,87] which is itself activated by Polo-like kinases to cleave Holliday junction-like structures when cells are preparing for mitosis [88]. Similarly at telomeres, Rad51- independent BIR may provide a final opportunity to elongate telomeres with arrested replication forks [73,74], using either single stranded regions of another telomere or extrachromosomal telomere circle. The fact that both are apparently independent of Rad51 but depend on Rad52 strongly suggests that these replication events are not necessarily initiated by strand invasion, but by strand annealing.

Taken together, the evidence from metazoan cells clearly implicates RDR in a multitude of fork recovery pathways and it is obvious that in many instances (for example a replication fork encountering a single strand break) this can initiate from a DNA - DSB and thus is formally equivalent to BIR as defined by the extensive analysis in budding yeast. However, RDR without an intervening DSB is also possible (see below) and it remains unclear how extensively these two pathways are used in human cells.

## 4 Recombination-dependent replication restart without a DSB

**Fig. 3:**
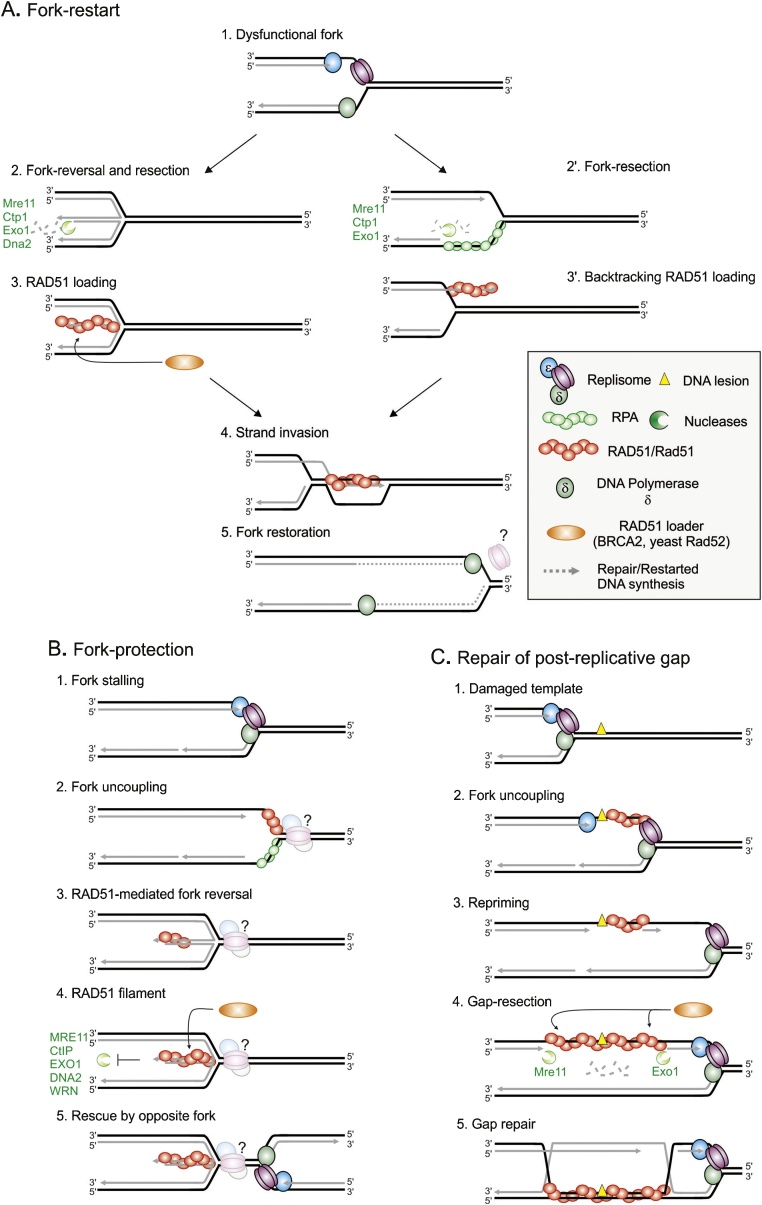
**Model of DSB-free recombination-dependent replication**. A. Restart of replication forks that have lost replication competence (1) requires recombination proteins. Several helicases and translocases can mediate fork reversal (2) allowing DNA-end resection to generate ssDNA on which Rad51 is loaded (3). The Rad51 filament then promotes strand invasion into the reformed parental DNA duplex to generate a D-loop intermediate (4) from which DNA synthesis is primed, restoring a functional but non-canonical fork (5). Alternatively, fork-restart may be initiated by resection of the lagging strand (2′) and the backtracking of the fork, generating an extruded leading strand onto which Rad51 is loaded (3′). In fission yeast, Rad51-mediated restarted forks are associated with a semi-conservative DNA synthesis during which both strands are synthetized by the DNA polymerase delta. B. Protection of stalled replication forks. Upon fork stalling (1), transient uncoupling allows RAD51 to bind to the ssDNA at the fork junction. This can occur independently of the BRCA2 loader (2). RAD51 promotes fork-reversal (3), possibly in a coordinated manner with additional helicases and translocases (*i.e.* SMARCAL1, ZRANB3, HTLF). BRCA2-dependent loading of RAD51 onto the fourth arm of the reversed fork protects the double-stranded DNA end from nucleolytic attack (4) by multiple exo- and endonucleases and helicases (*i.e.* DNA2, MRE11, CtIP, EXO1, WRN). Likely, RAD51-mediated fork reversal and protection maintain the integrity of the fork and allow appropriate merger with a converging forks (5). Whether limited end-resection is required for RAD51-mediated fork reversal and protection is unknown. The fate of the replisome at the reversed fork is also unknown. C. Repair of post-replicative gaps. When replicative DNA polymerases encounter a DNA lesion (1), transient and controlled uncoupling can occur, resulting in the formation of a ssDNA gap at the fork junction (2) which is subsequently coated by Rad51. DNA synthesis is resumed via repriming downstream the DNA lesion, leaving an internal ssDNA gap behind the moving fork (3). The ssDNA gap is enlarged by the DNA-end resection machinery facilitating further Rad51 loading by recombination mediators (4). The repair of the ssDNA gap is finalized in G2 phase (5).

The use of engineered Replication Fork Barriers (RFBs) has allowed a deeper understanding of the molecular transactions occurring at dysfunctional forks that act to both stabilize and restart them (Fig. 3). In the fission yeast *S. pombe*, the *RTS1* barrier has been used extensively to study the response to a dysfunctional replication fork [76,89,90]. In this system, Rad51-mediated fork restart occurs in ∼ 20 min and requires Rad52 and the strand exchange activity of Rad51 [34,69,91]. This supports the model in which Rad51 promotes the invasion of single stranded DNA with a 3′ end that acts to template restarted replication. Interestingly, in the *RTS1* system, a DSB is not induced at the site of the collapsed fork and the sister chromatids remain physically attached throughout the restart event [34,92,93]. Two models can be envisaged that mediate HR-dependent fork restart in this context (Fig. 3A): either the fork could reverse to form a DSB end that is homologous to the reannealed parental strands, or the fork could simply back-track, allowing annealing of the parental strands without the coordinated annealing of newly replicated strands. In this second scenario, nucleases would be required to remove the newly replicated lagging strand. In support of this, fission yeast Dna2 was shown to cleave nascent strands behind back-tracked forks when wild type cells are exposed to hydroxyurea, thus preventing reversed fork (chicken-foot) formation [94]. However, in these experiments the intra-S phase checkpoint was active, which maintains the vast majority of forks in a replication competent state. Whether Dna2 performs an equivalent task at dysfunctional forks has not been addressed.

**Fig. 4:**
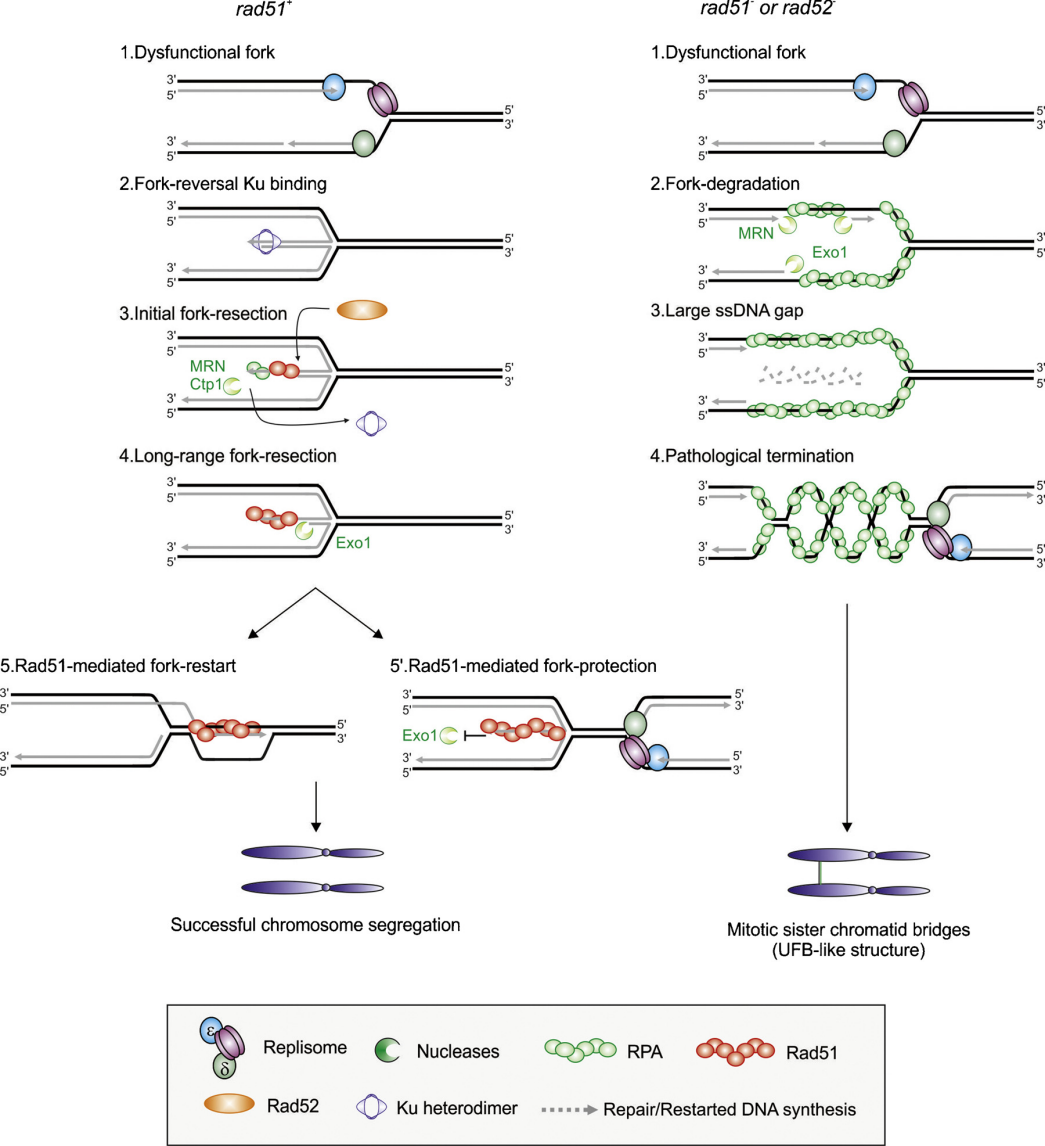
**Model of Rad51-mediated fork-protection and restart to avoid mitotic sister chromatid bridges in fission yeast**. A. A dysfunctional fork (1) undergoes fork-reversal providing a single double stranded DNA end for the non-homologous end joining heterodimer Ku to bind (2). Initial fork-resection mediated by Mre11/Rad50/Nbs1 (MRN) complex and Ctp1 (the fission orthologue of budding yeast Sae2 and mammalian CtIP), which remove Ku from the DSB end to generate short ssDNA gap onto which Rad51 can be loaded (3). The initial resection primes Exo1-mediated long-range resection to generate a larger ssDNA gap (4) onto which additional Rad51 is loaded. Rad51 promotes strand invasion into the parent duplex DNA, priming DNA synthesis and fork restoration (5). At the same time the Rad51 filament protects the reversed arm from extensive Exo1-dependent resection thus maintaining the fork structure in a form competent for merge with a converging fork (5′). Rad51-mediated fork protection and restart are genetically separable functions and the ultimate outcome depends strongly on the timing of the arrival of the converging fork. B. In the absence of Rad51 or Rad52, a dysfunctional fork (1) is not protected and undergoes uncontrolled resection by the DNA end-resection machinery (2). Large ssDNA gaps, up to 3 kb in size, form behind the fork (3) that are responsible for failure of termination of DNA replication (4). Such pathological termination events trigger mitotic sister chromatid bridges, a type of ultra-fine bridge that breaks during mitosis.

The formation of ssDNA that allows the formation of the Rad51 filament that is necessary to restart replication at the *RTS1* barrier was recently shown to be regulated in the same manner as resection is regulated to initiate DSB repair [92,93]. An initial short-range resection is mediated by the Mre11/Rad50/Nbs1 (MRN) complex and Ctp1 (the fission orthologue of CtIP). This generates a short ssDNA region of ∼100–150 bp that can prime long-range resection that is mediated by Exo1, generating larger ssDNA regions of up to 1KB (Fig. 4). Interestingly, the initial MRN-Ctp1-dependent resection is sufficient to initiate Rad51-mediated fork restart. Surprisingly, the initial resection was shown to be controlled by the Non Homologous End Joining (NHEJ) factor Ku, which binds to dysfunctional forks to prevent extensive degradation. We hypothesize that Ku recognizes and binds to the regressed arm of a reversed fork, from which it is subsequently removed by MRN and Ctp1. Thus, as described for DSB repair, the resection of a dysfunctional fork would occur via a two-step process, likely coordinating resection with Rad51 loading. Interestingly, without Rad51 or Rad52, this fork restart is prevented and the arrested forks are extensively resected directly by Exo1 [34] (Fig. 4). It is thus possible that Rad52-dependent Rad51 DNA loading generates a DNA structure that is obligatory for Ku recruitment, such as a reversed fork structure (see below) that acts as a key regulatory point in the control of resection. Alternatively, Rad51 and Rad52 may be involved in the active recruitment of the initial end-resection machinery. Consistent with this, mammalian RAD52 is required to prime MRE11-dependent resection of revered forks in the absence of BRCA2 [95].

Forks restarted at *RTS1* by HR without a DSB intermediate differ from origin-born replication forks [68,91,96]. Unlike forks initiated by BIR, they progress via semi-conservative DNA synthesis but with both strands being copied by polymerase delta [91] (Fig. 3A). Like BIR-initiated forks, forks restarted at *RTS1* are highly mutagenic: DNA synthesis is less processive, liable to replication slippage at micro-homology and highly prone to U-turn switches between inverted repeats that generate acentric and dicentric chromosomes [68,96]. Therefore, Rad51-mediated fork restart may contribute to the copy number variation observed in response to replication stress in human cells. In addition, during the act of restart, the newly replicated strands bound by Rad51 are able to search for homology throughout the genome and thus cause HR-dependent chromosomal rearrangements between repeat sequences [76,90].

Rad51 has multiple functions during replication, including the safeguarding of arrested forks from becoming dysfunctional, the protection of dysfunctional forks from excessive degradation (thus allowing their successful merger with converging forks (Fig. 3B) and the restart of replication either by BIR or in the absence of an induced DSB. In fission yeast, the ability of Rad51 to restart replication forks requires its strand exchange activity whereas its ability to protect dysfunctional forks from degradation requires only its DNA binding activity [34] (Fig. 4). It is therefore likely that the ability of the Rad51 filament to perform strand exchange in vivo is tightly regulated during the cell cycle to help maintain genome stability. In support of this, budding yeast Rad51, while associated with the fork during S-phase, repairs post replicative gaps only in G2 (Fig. 3C) and this repair function is regulated by CDK activity [97]. In mammals, the recombination-dependent activity of RAD51 at replication forks is regulated by RADX, a RPA-like single stranded DNA binding protein which antagonizes aberrant RAD51-dependent fork-remodeling [98].

## 5 Replication fork-protection and remodeling towards fork-reversal

**Fig. 5:**
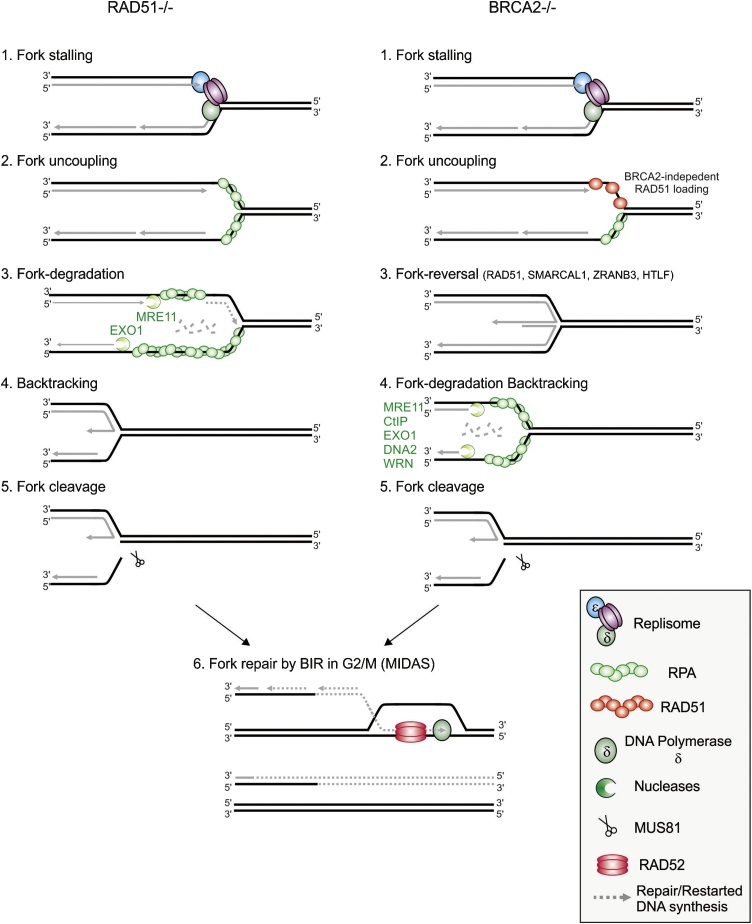
**Abnormal replication intermediates in the absence of recombination factors**. A. Without RAD51. Replication fork stalling (1) results in uncoupling and ssDNA gap formation at the fork junction (2). Resection of the ssDNA gap generates large stretched of ssDNA which cannot be repaired without RAD51 (3). Backtracking of the resected-fork (4) potentially provides a substrate to MUS81 (5). Fork-repair can subsequently be initiated in mitosis by RAD52 (6). B. Without BRCA2. Replication fork stalling (1) results in uncoupling and ssDNA gap formation. RAD51 binds independently of BRCA2 (2) and fork reversal is mediated by RAD51 cooperatively with helicases and translocases (*i.e.* SMARCAL1, ZRANB3, HTLF) (3). The reversed fork provides an entry point for the uncontrolled activity of the DNA end-resection machinery, resulting in a large ssDNA gap (4). As in (A), backtracking and fork cleavage can facilitate fork-repair in mitosis (5 and 6).

While the requirement of HR for efficient unperturbed DNA replication could be explained by a necessity for DSB repair of replication-dependent DNA lesions, several papers at the start of the decade identified links between replication and HR that were independent of direct DNA repair in metazoan, as proposed in yeast [76]. Hashimoto [99] used Xenopus replicating extracts and electron microscopy to show that, in the absence of RAD51, forks accumulated increased levels of MRE11-dependent gaps in the newly synthesized DNA behind the fork and elevated levels of leading and lagging strand uncoupling at the fork junction (these latter being Mre11-independent) (Fig. 5). Petermann et al, [78] used DNA combing following short-term (1–2 h) and long-term (12–14 h) hydroxyurea exposure to show that RAD51 promoted the resumption of DNA synthesis following short-term inhibition. Only the longer-term inhibition of replication correlated with the formation of DSBs, which are likely to be associated with the activity of Mus81 [100,101].

These functions for HR proteins in protecting replication forks from the accumulation of DNA lesions following short-term replication arrest and preventing forks from becoming dysfunctional (or rapidly restarting them if they did) were extended by the work of Schlacher et al, [102,103]. These studies used DNA fiber analysis to show that, when replication was inhibited by hydroxyurea in BRCA2 deficient cells, newly replicated DNA was rapidly (within 15 min) degraded at a rate of between 1.8–2.2 kb per hour in an Mre11-dependent manner (Fig. 3B). This protection of forks from degradation required Rad51 filament formation plus filament stabilization by BRCA2 [102] and the core Fanconi anaemia pathway [103]. Because Fanconi anaemia cell lines displayed severe fork de-protection phenotypes but can undergo classical DSB repair, it was proposed that fork protection defined a novel "recombination independent" function for HR, where the formation of a RAD51 filament protected ssDNA rather than promoting strand exchange (Fig. 5).

Despite the fact that Fanconi anaemia and BRCA2-defective cells are defective in fork protection (as defined by the inability to prevent nascent strand degradation), they do not show a reduced ability to restart transiently stalled forks or display significant sensitivity to transient replication arrest [102,103]. However, they do show an increase in genome instability after such treatments, manifesting chromatid breaks and radial structures on mitotic chromosome spreads. This contrasts with cells defective in the BLM helicase, which show a decrease in fork resumption and an increase in sensitivity to fork-stalling agents [104], despite not being defective for fork protection [103]. Thus, protection of forks by HR proteins, which prevents nascent strand degradation and the appearance of single stranded gaps by electron microscopy analysis, does not directly equate to preventing forks becoming dysfunctional and suggested that RAD51 plays multiple and separable roles in regulating replication.

It remains unclear why the loss of fork protection is detrimental to genome integrity following replication stress: conceptually, unprotected forks could be efficiently rescued by converging forks. Perhaps an explanation can be extrapolated from a recent study in fission yeast. This showed that dysfunctional forks arrested at *RTS1* in the absence of Rad51 accumulate large ssDNA gaps and that, despite the presence of a converging fork, these unprotected forks are converted into sister chromatid bridges during mitosis, which subsequently break [34] (Fig. 4). This demonstrated that the lack of Rad51 impaired appropriate fork merging. This role of Rad51 in promoting fork merging was dependent on its ability to bind DNA and to form a filament, but not on the ability to engage in strand exchange, a situation reminiscent of fork protection described by Schlacher et al. [102]. Thus, while these arrested forks in the fission yeast were dysfunctional, the ssDNA associated with unprotected, but otherwise functional, forks in human cells could cause a similar failure of fork merging: RAD51, BRCA2 and the Fanconi anaemia pathways may hence function to maintain transiently arrested forks in a conformation that is competent for termination. Consistent with this, linear DNA harboring a ssDNA overhang bound by RAD51 filament (but not when bound by RPA) is resistant to MRE11-mediated degradation in vitro [105]. In light of the complexity of the function of HR factors during DNA replication, it remains to be defined which BRCA2 and HR functions are critical to suppress tumor initiation.

A specific DNA structure at replication forks, known as a reversed fork or chicken-foot, has emerged as a pivotal intermediate during the response to replication stress of metazoan cells ([106,107] (reviewed in [108]). At reversed forks, the newly replicated strands are annealed together in a manner that is coordinated with the re-annealing of the parental strands, forming a four-way structure equivalent to a Holliday junction (Fig. 3B). Fork-reversal was initially proposed as a DNA configuration that allowed DNA damage tolerance by translesion DNA synthesis [109]. Physical evidence for reversed forks in eukaryotic cells was first provided by their visualization by electron microscopy: checkpoint-defective mutants of the yeast *S. cerevisiae* that were dying from unresolved replication stress showed high levels of reversed fork structures, but these were not seen in wild-type cells exposed to equivalent stress [110]. This initially led the hypothesis that fork reversal represented pathological "dead-end" DNA structures at terminally arrested forks. However, subsequent studies established that fork reversal occurs at high frequency upon reversible replication stress in numerous biological systems, including mammalian cells [106] and physarum polycephalum [111]. They could also be induced non-pathologically in the fission yeast *S. pombe* [94]. A broad spectrum of intrinsic and exogenous replication stresses are now known to promote the extensive formation of reversed forks in many organisms. These include dNTP depletion, various DNA damaging agents, topoisomerase inhibitors, intrinsically difficult to replicate sequences (such as tri-nucleotide repeats) and oncogene expression causing unbalanced DNA replication. Reversed forks have also been observed in rapidly divided embryonic stem cells [31,112].

A high frequency of fork reversal therefore appears to be a conserved cellular response to replication stress. Likely, forks encountering obstacles rapidly undergo controlled fork reversal but remain fully capable of resuming DNA replication. Indeed, it cannot be excluded that forks which have not been physically impeded in their progression could undergo fork reversal as a consequence of a global cellular response acting in *cis*. Fork reversal appears to provide a way to prevent the degradation of newly synthesized DNA and the associated accumulation of lesion that can result in genetic instability [71,95,105,113]. This would not conflict with the original hypothesis, where the annealing of the two newly synthesized strands can also provide the opportunity for DNA damage tolerance by translesion synthesis. Furthermore, the annealing of the parental strands may provide the opportunity to remove the original problem by providing, for example, the correct context for repairing base lesions. To what extent fork reversal is an obligatory DNA intermediate during the resumption of replication by an otherwise competent fork remains unclear. In mammalian cells, the RecQ-like helicase RecQL1 has the ability to migrate reversed forks to resolve the reversed fork structure and allow replication to be resumed [114]. Alternatively, the regressed arm of a functional but reversed fork may be fully resected by nuclease activities, such as DNA2 as has been proposed in fission yeast [94] and humans [115]. The fate of the replisome during fork reversal is unknown and the exact role that the reversed fork structure plays in handling replication dynamics therefore remains to be defined.

When forks are reversed, they are protected from extensive degradation by the stabilization of RAD51 filaments by BRCA2. In the absence of BRCA2, reversed forks are rapidly degraded, resulting in nascent strand loss (fiber analysis) and a reduction in the steady-state level of reversed forks upon electron microscopy (Fig. 5). Surprisingly, the initial fork-reversal in both BRCA2 proficient and deficient cells is also dependent on RAD51 [71,95]. This function in initiating fork reversal can be genetically separated from the function of RAD51 in protecting reversed forks: a mutant of RAD51-T131P that results in unstable RAD51 filaments is able to promote reversed forks (in both BRCA2 proficient and deficient hydroxyurea treated cells) but is unable to protect reversed forks from subsequent degradation when BRCA2 is present [95,116]. Thus, RAD51 has a BRCA2-independent function in promoting fork reversal followed by a BRCA2-dependent function in protecting the reversed fork from excessive degradation (Fig. 5). Consistent with a stable filament-independent role for Rad51 at replication forks that is independent of BRCA2, a fraction of Rad51 can associate with chromatin undergoing replication in the absence of BRCA2 [105,117]. Potentially, Rad51 associates with replication forks even in the absence of stress to facilitate fork progression through intrinsic fork obstacles. In fission yeast, Rad51 has been reported to associate directly with the CMG helicase [118] and, in budding yeast, Rad51 association with the replication fork is a prerequisite to ensure the later repair of damage induced-ssDNA gaps left behind the moving fork [97]. A physical association between the DNA polymerase alpha and Rad51 has also been suggested to facilitate leading-strand re-priming downstream of DNA lesions to ensure continuous fork progression [105]. Thus, there is ample prescient for recombination-independent functions for Rad51 during DNA replication.

In addition to occurring at functionally competent forks, fork reversal is likely to accompany dysfunctional forks that cannot resume replication without the intervention of the HR machinery. A potential benefit of fork reversal in this situation is the formation of a structure (a DSB end) that can provide an entry point for the resection machinery (Fig. 4) [93,115]. This provides an opportunity to regulate the production of a 3′ ssDNA that can be used to establish a D-loop on the reannealed parental strands that can prime DNA synthesis if the dysfunctional fork is not rescued by a converging fork. Indeed, in fission yeast the requirement for Ku in the regulation of resection at dysfunctional forks has led to the proposal that fork reversal is instrumental during Rad51-mediated restart of dysfunctional forks. The formation of a reversed fork may be important for the coordination of the resection with fork restart by homologous recombination (see above). It remains unclear if the lack of visualization of reversed fork structures by electron microscopy in wild type yeast cells subjected to replication stress is a consequence of fork reversal not being a common response to replication perturbation in simple eukaryotic organisms, or if these structures are frequently formed but rapidly resolved.

It again remains unclear if fork reversal to provide a regressed double stranded arm is an obligatory step in restarting a dysfunctional fork. As discussed above, an alternative would be to degrade the nascent lagging strand while displacing the nascent leading strand and reannealing the two parental strands (Fig. 3A). Nonetheless, once a regressed arm has formed, experiments in fission yeast suggest that its controlled resection is necessary to ensure restart. Alternative models for fork restart from a reversed fork propose that MUS81 resolves the Holliday junction to generate a single-ended DSB [71,72,119,120]. While single ended breaks generated in S phase by replication of a persisting ssDNA nick (as in BIR, see above) is clearly permissive to restarting replication, it is unclear if MUS81-dependent cleavage of arrested forks is a major pathway of fork restart by HR, either in yeast or in human cells. Petermann et al. [78] suggested such breaks only appear in human cells after extensive (24 h) incubation in hydroxyurea and that these DSBs are not the initiating point for subsequent replication: replication occurring after release from 24 h hydroxyurea arrest instead initiated from new origin firing. There is evidence of MUS81-dependent breaks and associated replication restart in BRCA2 deficient cells (but intriguingly not in BRCA1-deficient cells) that have been treated with hydroxyurea for 2 h [71], but at present there is insufficient evidence to support MUS81-dependent breaks forming as a physiological response to fork arrest, or to determine if such breaks engage in BIR.

## 6 Concluding remarks

The resistance of BRCA-deficient tumors to chemotherapy treatments, including PARP inhibitors, is closely associated with restoration of replication fork protection, prompting a significant effort to understand how replication fork dynamics impacts on cancer treatments [121,122]. Numerous additional proteins have been shown to mediate fork reversal and thus potentially restrain MRE11 activity at reversed forks including BRCA1, RAD54, REV1, RECQL5, PARP1 [122], FBH1 (a conserved Uvrd-family 3′-5′ DNA helicases) [123] FancM (Fanconi anemia 3′-5′ DNA helicase) (reviewed in [107]). Whether the fork-reversal activities of specific factors are related to specific features of stalled and arrested forks that generated by distinct barriers, or whether they have largely redundant activities at all arrested forks remains unknown. It is known that some factors travel with the replication machinery and, in vitro, some (such as SMARCAL1, ZRANB3 and HTLF) recognize different fork structures and have differential requirements for RPA-bound ssDNA gap to promote in vitro helicase/translocase activities.

Several other proteins have been implicated in the inhibition of resection factors acting downstream of MRN, DNA2 (BOD1L) and EXO1 (WRN) [124]. Many additional proteins have been implicated either directly or indirectly in fork remodeling. These including SWI/SNF helicase-like proteins such as yeast Rad5, its human orthologue HLTF and a range of potential chromatin-associated factors suggesting that fork dynamics is tightly controlled as an important nexus for the regulation of replication. Understanding how replication fork dynamics are regulated in response to various type of replication stress and during cell cycle progression is critical not only to understand how cells achieve the faithful duplication of their genome, but also to define better anti-cancer therapeutics.
